# Autosomal recessive nonsyndromic hearing impairment in two Finnish families due to the population enriched *CABP2* c.637+1G>T variant

**DOI:** 10.1002/mgg3.1866

**Published:** 2022-02-11

**Authors:** Thashi Bharadwaj, Isabelle Schrauwen, Anushree Acharya, Liz M. Nouel‐Saied, Marja‐Leena Väisänen, Minna Kraatari, Elisa Rahikkala, Irma Jarvela, Jouko Kotimäki, Suzanne M. Leal

**Affiliations:** ^1^ Center for Statistical Genetics Gertrude H. Sergievsky Center, and the Department of Neurology Columbia University Medical Center New York NY USA; ^2^ Northern Finland Laboratory Centre NordLab and Medical Research Centre Oulu University Hospital and University of Oulu Oulu Finland; ^3^ Department of Clinical Genetics PEDEGO Research Unit and Medical Research Center Oulu Oulu University Hospital and University of Oulu Oulu Finland; ^4^ Institute of Biomedicine University of Turku Turku Finland; ^5^ Department of Medical Genetics University of Helsinki Helsinki Finland; ^6^ Department of Otorhinolaryngology Kainuu Central Hospital Kajaani Finland; ^7^ Taub Institute for Alzheimer’s Disease and the Aging Brain Columbia University Medical Center New York NY USA

**Keywords:** autosomal recessive, CABP2, hearing impairment

## Abstract

**Background:**

The genetic architecture of hearing impairment in Finland is largely unknown. Here, we investigated two Finnish families with autosomal recessive nonsyndromic symmetrical moderate‐to‐severe hearing impairment.

**Methods:**

Exome and custom capture next‐generation sequencing were used to detect the underlying cause of hearing impairment.

**Results:**

In both Finnish families, we identified a homozygous pathogenic splice site variant c.637+1G>T in *CAPB2* that is known to cause autosomal recessive nonsyndromic hearing impairment. Four *CABP2* variants have been reported to underlie autosomal recessive nonsyndromic hearing impairment in eight families from Iran, Turkey, Pakistan, Italy, and Denmark. Of these variants, the pathogenic splice site variant c.637+1G>T is the most prevalent. The c.637+1G>T variant is enriched in the Finnish population, which has undergone multiple bottlenecks that can lead to the higher frequency of certain variants including those involved in disease.

**Conclusion:**

We report two Finnish families with hearing impairment due to the *CABP2* splice site variant c.637+1G>T.

## Graphical abstract

The genetic architecture of hearing impairment in Finland is largely unknown. Here, we investigated two Finnish families with autosomal recessive nonsyndromic symmetrical moderate‐to‐severe hearing impairment. Exome and custom capture next‐generation sequencing were used to detect the underlying cause of hearing impairment. In both Finnish families, we identified a homozygous pathogenic splice site variant c.637+1G>T in *CAPB2* that is known to cause autosomal recessive nonsyndromic hearing impairment.

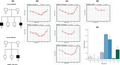

## 1 INTRODUCTION

Over 70 autosomal recessive (AR) nonsyndromic (NS) hearing impairment (HI), genes have been identified, including *CABP2* (OMIM: 607314) that encodes Ca^2+^‐binding protein (CaBP) 2. It belongs to the CaBP family that is related to calmodulin and is expressed in the cochlea and hair cells. CABP2 facilitates normal functioning of the peripheral auditory system by inhibiting Ca_V_1.3 Ca^2+^‐channel inactivation (Cui et al., 2007; Picher et al., 2017; Yang et al., 2018). Four different *CABP2* variants have been reported to cause ARNSHI (DFNB93) (Table 1). For the Finnish population within the Genome Aggregation Database (gnomAD) (Karczewski et al., 2020), *GJB2* has the most frequent ARNSHI pathogenic variants, followed by *CABP2* (NM_016366.3) c.637+1G>T (allele frequency [AF] = 3.3 × 10^−3^). *CABP2* has not yet been reported to be involved in ARNSHI in Finland. We report on two Finnish families with ARNSHI due to the splice site variant c.637+1G>T. This variant is classified as pathogenic according to the American college of medical genetics (ACMG) guidelines. The Finnish population has a unique genetic architecture in which certain variants are enriched while the variant diversity is reduced compared to other populations (Chakchouk et al., 2019; Chheda et al., 2017).

**TABLE 1:** Description of families with pathogenic or likely pathogenic CABP2 variants

# Fam	Country	Variant	Onset	Bilateral	Severity	Consang	References
3	Iran	c.637+1G>T^1^	Prelingual	Yes	Moderate to severe	Yes	Schrauwen et al. (2012)
1	Turkey	c.490‐1G>T	Prelingual	Yes	NI	NI	Bademci et al. (2016)
1	Italy	c.466G>T^2^	Prelingual	Yes	Moderate to Severe	Yes	Picher et al. (2017)
1	Iran	c.311G>A^3^	Prelingual	Yes	Severe	Yes	Koohiyan et al. (2019)
1	Pakistan	c.637+1G>T	NI	NI	Severe to profound	Yes	Richard et al. (2019)
1	Denmark	c.637+1G>T	Prelingual	Yes	Moderate	No	Sheyanth et al. (2021)
2	Finland	c.637+1G>T	Prelingual^4^	Yes	Moderate to severe	No	This study

Abbreviations: # Fam, Number of Families; Consang, Consanguineous; NI, not indicated.

^1^ Amino acid changes: p. Phe164Serfs*4.

^2^ p.(Glu156*).

^3^ p.(Gly104Asp).

^4^ For one child prelingual onset could not be confirmed.

## 2 METHODS

### 2.1 Familial and clinical examination

The study was approved by the ethics committee of the Oulu University Hospital (EETTMK: 55/2018 and 186/2020) and from the institutional review board (IRB) Columbia University (IRB‐AAAS2343). Two children in family FINHEAR1 and one child in family FINHEAR2 with HI were born to parents of Finnish origin from the Kainuu and Central Ostrobothnia regions, respectively. No other family members were reported to be hearing impaired. Available medical records as well as familial and clinical history were evaluated. Physical examination, transient otoacoustic emissions (TEOAE), and pure‐tone audiometry were performed at 125–8000 Hz for the affected children of FINHEAR1. TEOAE, auditory steady‐state response (ASSR), and auditory brainstem response (ABR) were evaluated for the affected child of FINHEAR2. DNA samples were obtained from the unaffected parents and their affected children.

### 2.2 Exome sequencing and data analysis

**FIGURE 1:**
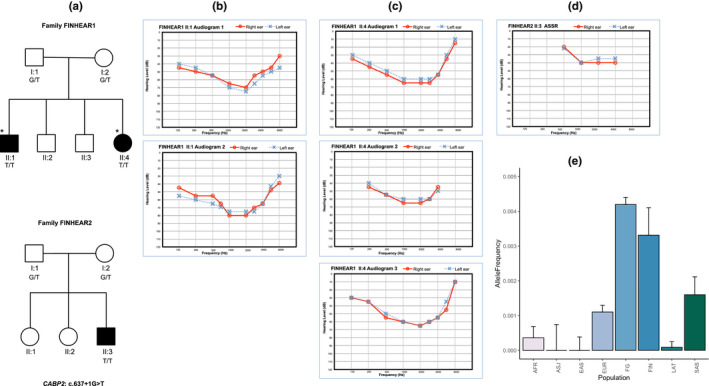
Pedigree drawing and Diagnostic test results. Panel A: Pedigrees and segregation of the *CABP2* (NM_016366.3) c.637+1G>T variant in FINHEAR1 and FINHEAR2. A star indicates the family members whose DNA samples underwent exome sequencing. Circles represent females and squares represent males, those individuals with solid symbols have HI while those with clear symbols are unaffected. The *CABP2* c.637+1G>T genotype is indicated under each family member. Panel B: Air conduction thresholds for FINHEAR1 II:1 at 6 years of age (Audiogram 1) and 13.7 years of age (Audiogram 2). Circles with smooth connecting lines in red represent the right ear and crosses with dotted connecting lines in blue represent the left ear. Panel C: Air conduction thresholds for FINHEAR1 II:4 at 2.4 years of age (Audiogram 1), 3.0 years of age (Audiogram 2), and 3.5 years of age (Audiogram 3). Circles with smooth connecting lines in red represent the right ear and crosses with dotted connecting lines in blue represent the left ear. Panel D: Auditory steady‐state response (ASSR) for FINHEAR2 II:3 recorded at 4 months of age revealed responses on average at 40–55 dB levels indicating moderate sensorineural HI. Circles with smooth connecting lines in red represent the right ear and crosses with dotted connecting lines in blue, the left ear. II.3 is too young to obtain audiograms. Panel E: Comparison of allele frequencies of *CABP2*: c.637+1G>T. Allele frequencies for the *CABP*2: c.637+1G>T in various populations were obtained from gnomAD. The error bars for each population denote the upper 95% confidence interval. *CABP*2: c.637+1G>T has the highest frequency in the Finnish population and was absent in Ashkenazi Jews and East Asians. The difference in allele frequency between the Finnish and each gnomAD population is statistically significant with the largest *p* value occurring for the comparison with non‐Finnish Europeans (*p* = 3.6 × 10^−5^). Abbreviations and allele frequencies: African/African‐American (AFR) (3.6 × 10^−4^), Ashkenazi Jewish (ASJ) (0), East Asian (EAS) (0), European‐non‐Finnish (EUR) (1.1 × 10^−3^), European‐Finnish (FIN) (3.3 × 10^−3^), FinnGen‐Finnish (FG) (4.2 × 10^−3^) Latino/Admixed American (LAT) (8.6 × 10^−5^), and South Asian (SAS) (1.6 × 10^−3^)

Genomic DNAs from the affected members of FINHEAR1 and FINHEAR2 were screened for pathogenic ARNSHI variants in the coding exon of *GJB2* via Sanger sequencing. DNA samples from II:1 and II:4 of family FINHEAR1 and II:3 of family FINHEAR2 (Figure 1a) underwent sequencing using exome and a custom capture array, respectively. For FINHEAR1, library construction and exon capture were completed using BGISEQ/Agilent SureSelect Human All Exon V6 kit (60.5 Mb) with an average‐sequencing read depth of 109.35× for II:1 and 97.37× for II:4. For FINHEAR2, a clinical custom capture 95 NSHI gene panel (Illumina TruSight One Expanded sequence panel) was used (see Table S1 for a list of the genes). An average sequence read depth of 100× was obtained for II:3. Read alignment, variant calling, annotation, and filtering were performed as previously described (Schrauwen et al., 2020). The variants were validated and tested for segregation by Sanger sequencing using DNA samples from the affected members and unaffected parents.

## 3 RESULTS

FINHEAR1 has two affected family members, II:1 and II:4 (Figure 1a). II:1 initially passed TEOAE newborn hearing screening. He was seen again at 6 years of age due to purulent otitis media and suspected HI. Observations during the operative procedures (tympanostomy and adenotomy) indicated chronic otitis. HI persisted even after otitis media treatment and resolution. HI was confirmed by pure‐tone audiometry at 6 years of age (Figure 1b). For II:4, bilateral HI was detected at 17 days after birth through TEOAE newborn screening. Audiograms taken at the ages of 6.0 and 13.7 years for II:1 and at 2.4, 3.0, and 3.5 years for II:4 (Figure 1c) feature symmetrical, moderate‐to‐severe non‐progressive HI that is more conspicuous in the mid‐frequencies forming a U‐shaped audiogram (Figure 1b,c). FINHEAR2 has one affected member, II:3 (Figure 1a), who failed newborn screening as otoacoustic emissions were not elicited. At 4 months of age, TEOAE showed no responses, and ASSR (Figure 1d) and ABR results revealed responses on average at 40–55 dB levels which led to a diagnosis of moderate sensorineural HI. For neither family was there any indications that HI was part of a syndrome.

The only rare homozygous variant which was observed in the sequence data was the splice site *CABP2* c.637+1G>T variant (Table S2). Additionally, no potential compound heterozygous variants were observed. The c.637+1G>T variant is enriched in the Finnish population (Figure 1e). Although six of the eight previously published families with *CABP2*‐related HI were consanguineous, the parents in these Finnish families reported to be unrelated as far as they know.

## 4 DISCUSSION

*CABP2*‐related ARNSHI has been reported in families from Iran (*N* = 4) (Koohiyan et al., 2019; Schrauwen et al., 2012), Turkey (*N* = 1) (Bademci et al., 2016), Italy (*N* = 1) (Picher et al., 2017), Pakistan (*N* = 1) (Richard et al., 2019), and Denmark (*N* = 1) (Sheyanth et al., 2021). For five of these families, the *CABP2* (NM_016366.3) c.637+1G>T is the causative variant (Iranian [*N* = 3], Pakistani [*N* = 1], and Danish [*N* = 1]) (Table 1). For FINHEAR1, the HI presents with the classic audiograms observed for *CABP2* variants. Although FINHEAR1 II:4 presented with congenital HI, we cannot confirm her brother II:1 has congenital HI since he was not diagnosed until 6 years of age.

The c.637+1G>T variant is suggested to result in skipping of exon 6 which in turn is predicted to cause a shifted reading frame and a truncated protein in the absence of RNA degradation via nonsense‐mediated mRNA decay. Functional studies demonstrate that the truncation impairs but does not completely obliterate its ability to modulate Ca_v_1.3 (Picher et al., 2017; Schrauwen et al., 2012).

The AF of the *CABP2* c.637+1G>T variant within the Finnish population is 3.3 × 10^−3^ (*N* = 12,516) (95% confidence interval [CI] 2.6 × 10^−3^, 4.1 × 10^−3^) in gnomAD with no homozygous individuals. FinnGen (2021), which includes genotype and imputed genome data obtained on 218,792 individuals, the imputed c.637+1G>T variant has an AF of 4.2 × 10^−3^ (95% CI 4.0 × 10^−3^, 4.4 × 10^−3^). There are four individuals who are likely homozygous for the imputed c.637+1G>T variant. FinnGen reports no association between the c.637+1G>T variant and sensorineural hearing loss (15,952 cases and 196,592 controls) (*p* value = 4.4 × 10^−1^). Although this variant is enriched in the Finnish population, *CABP2* is likely a rare cause of HI in Finland. The estimated prevalence of HI in Finland due to homozygous c.637+1G>T variants ranges from 0.7 to 1.9 per 100,000 individuals. As the allelic spectrum of pathogenic *CABP2* variants in Finland is unknown, the frequency of HI due to *CABP2* could be higher if there are other yet to be identified causal variants.

The Finns are an isolated population and their genetic architecture has been molded by several ancient and recent migratory and multiple bottleneck events as well as rapid population growth in the last 10 generations (Chheda et al., 2017). These events have shifted proportion and frequency of variants and contributed to decreased genetic diversity. The history of this population may have caused a higher prevalence of 36 monogenic Mendelian disorders in Finland compared with the rest of the world. These Mendelian diseases include epilepsy, muscular dystrophy, and Usher syndrome and are classified as Finnish heritage diseases (Norio, 2003).

In conclusion, we identified the *CABP2* c.637+1G>T variant that segregates with moderate‐to‐severe ARNSHI in two families of Finnish origin. To the best of our knowledge, these are the first reported cases of *CABP2*‐related ARNSHI in Finland.

## CONFLICT OF INTEREST

All authors declare no conflict of interest.

## AUTHOR CONTRIBUTIONS

S.M.L. developed the concept of the study. S.M.L., I.S., M‐L.V., M.K., E.R., I.J., and J.K. organized and coordinated the study. M‐L.V., M.K., E.R., I.J., and J.K. gathered the clinical information. I.S., T.B., and A.A. analyzed the data. A.A. and L.M.N‐S conducted molecular experiments. T.B. wrote the first draft of the manuscript. S.M.L and I.S. reviewed and revised the manuscript. All authors have agreed to the final version of the manuscript.

## Supporting information

Table S1

Table S2

## Data Availability

The data that support the findings of this study are available from the corresponding author upon request. The variant has been submitted to ClinVar database at https://www.ncbi.nlm.nih.gov/clinvar/ with accession number SCV001622777.1.
